# Supplementary material for: Soluble tumor necrosis factor receptor 2 is associated with progressive diabetic kidney disease in patients with type 2 diabetes mellitus
Source: PLoS One. 2022 Apr 12;17(4):e0266854. doi: 10.1371/journal.pone.0266854 (PMC9004780; doi:10.1371/journal.pone.0266854)
Supplement: S1 Table — (DOCX) [file pone.0266854.s001.docx]

| **Table S1.** Baseline characteristics and renal composite events of patients grouped according to soluble tumor necrosis factor receptor type 2 (sTNFR2) concentrations | | | |  |
| --- | --- | --- | --- | --- |
|  | sTNFR2 | |  |  |
|  | ≥1.608 ng/mL  (n = 197) | <1.608 ng/mL  (n = 167) | P value |  |
| Age | 65.2±11.7 | 56.9±12.6 | <0.001 |  |
| Male sex (%) | 133 (67.5) | 123 (73.7) | 0.201 |  |
| Smoking (%) | 57 (28.9) | 54 (32.3) | 0.482 |  |
| Coronary artery disease (%) | 45 (22.8) | 27 (16.2) | 0.111 |  |
| Hyperlipidemia (%) | 163 (82.7) | 132 (79.0) | 0.370 |  |
| Hypertension (%) | 130 (66.0) | 94 (56.3) | 0.058 |  |
| Retinopathy (%) | 24 (12.2) | 10 (6.0) | 0.043 |  |
| Neuropathy (%) | 23 (11.7) | 10 (6.0) | 0.060 |  |
| Albuminuria (%) | 81 (41.1) | 40 (24.0) | 0.001 |  |
| CKD stage 3 (%) | 56 (28.4) | 11 (6.6) | <0.001 |  |
| CKD stage 3 with albuminuria (%) | 33 (16.8) | 8 (4.8) | <0.001 |  |
| Duration of diabetes (year) | 12.5±7.8 | 10.7±7.1 | 0.020 |  |
| Body mass index | 25.9±4.7 | 26.8±4.1 | 0.064 |  |
| Waist-hip ratio | 0.93±0.06 | 0.94±0.07 | 0.226 |  |
| Systolic blood pressure (mmHg) | 133.5±15.8 | 132.0±14.9 | 0.342 |  |
| Diastolic blood pressure (mmHg) | 77.4±11.2 | 77.1±10.0 | 0.792 |  |
| Mean blood pressure (mmHg) | 114.8±13.1 | 113.7±11.9 | 0.395 |  |
| Urinary albumin-creatinine ratio (mg/g Cr) | 151.7±379.1 | 49.5±154.3 | 0.001 |  |
| HbA1C (%) | 7.1±0.8 | 7.1±0.9 | 0.706 |  |
| Total cholesterol (mg/dL) | 167.4±29.6 | 166.1±26.3 | 0.687 |  |
| Creatinine (mg/dL) | 1.03±0.31 | 0.89±0.19 | <0.001 |  |
| eGFR (mL/min/1.73m^2^) | 71.8±19.3 | 85.8±16.7 | <0.001 |  |
| sTNFR2 (ng/mL) | 2.78±1.07 | 1.09±0.26 | NA |  |
| Metformin (%) | 148 (75.1) | 135 (80.8) | 0.192 |  |
| Sulfonylurea (%) | 76 (38.6) | 68 (40.7) | 0.677 |  |
| Dipeptidyl peptidase-4 inhibitor (%) | 39 (19.8) | 29 (17.4) | 0.553 |  |
| Sodium/glucose co-transporter 2 inhibitor (%) | 3 (1.5) | 10 (6.0) | 0.022 |  |
| Insulin (%) | 34 (17.3) | 30 (18.0) | 0.860 |  |
| Renin-angiotensin system blockade (%) | 109 (55.3) | 65 (38.9) | 0.002 |  |
| Diuretics (%) | 28 (14.2) | 15 (9.0) | 0.123 |  |
| Renal composite events (%) | 55 (27.9) | 18 (10.8) | <0.001 |  |
| eGFR decline > 30% (%) | 27 (13.7) | 11 (6.6) | 0.027 |  |
| Worsening albuminuria (%) | 36 (18.3) | 11 (6.6) | 0.001 |  |

Data are expressed as mean±SD for continuous variables and numbers and percentages for non-continuous variables. Differences between groups stratified by the cut-off value of sTNFR2 were analyzed by Student's t-test for continuous variables and Pearson's chi-squared test for non-continuous variables.

CKD, chronic kidney disease; Cr, creatinine; HbA1C, hemoglobin A1C; eGFR, estimated glomerular filtration rate; sTNFR2, soluble tumor necrosis factor receptor type 2; NA, not applicable.
